# Immunoglobulin Kappa C Predicts Overall Survival in Node-Negative Breast Cancer

**DOI:** 10.1371/journal.pone.0044741

**Published:** 2012-09-28

**Authors:** Zonglin Chen, Aslihan Gerhold-Ay, Susanne Gebhard, Daniel Boehm, Christine Solbach, Antje Lebrecht, Marco Battista, Isabel Sicking, Christina Cotarelo, Cristina Cadenas, Rosemarie Marchan, Joanna D. Stewart, Mathias Gehrmann, Heinz Koelbl, Jan G. Hengstler, Marcus Schmidt

**Affiliations:** 1 Department of Obstetrics and Gynecology, Johannes Gutenberg University, Mainz, Germany; 2 Department of Medical Biometry, Epidemiology and Informatics, Johannes Gutenberg University, Mainz, Germany; 3 Department of Pathology, Johannes Gutenberg University, Mainz, Germany; 4 Leibniz Research Centre for Working Environment and Human Factors (IfADo), Dortmund University of Technology, Dortmund, Germany; 5 Bayer GmbH, Leverkusen, Germany; Sudbury Regional Hospital, Canada

## Abstract

**Background:**

Biomarkers of the immune system are currently not used as prognostic factors in breast cancer. We analyzed the association of the B cell/plasma cell marker immunoglobulin kappa C (IGKC) and survival of untreated node-negative breast cancer patients.

**Material and Methods:**

IGKC expression was evaluated by immunostaining in a cohort of 335 node-negative breast cancer patients with a median follow-up of 152 months. The prognostic significance of IGKC for disease-free survival (DFS) and breast cancer-specific overall survival (OS) was evaluated with Kaplan-Meier survival analysis as well as univariate and multivariate Cox analysis adjusted for age at diagnosis, pT stage, histological grade, estrogen receptor (ER) status, progesterone receptor (PR) status, Ki-67 and human epidermal growth factor receptor 2 (HER-2) status.

**Results:**

160 patients (47.7%) showed strong expression of IGKC. Univariate analysis showed that IGKC was significantly associated with DFS (P = 0.017, hazard ratio [HR] = 0.570, 95% confidence interval [CI] = 0.360–0.903) and OS (P = 0.011, HR = 0.438, 95% CI = 0.233–0.822) in the entire cohort. The significance of IGKC was especially strong in ER negative and in luminal B carcinomas. In multivariate analysis IGKC retained its significance independent of established clinical factors for DFS (P = 0.004, HR = 0.504, 95% CI = 0.315–0.804) as well as for OS (P = 0.002, HR = 0.371, 95% CI = 0.196–0.705).

**Conclusion:**

Expression of IGKC has an independent protective impact on DFS and OS in node-negative breast cancer.

## Introduction

For many years researchers have tried to characterize prognostic factors, but have only made limited progress [1]. Predicting the prognosis of patients still relies largely on traditional prognostic factors such as age, pT stage and histological grade. Gene-based testing like Oncotype DX, Endopredict or Mamma Print is increasingly used to determine prognosis [2]–[4]. However, these gene-expression arrays rely largely on proliferation and estrogen receptor (ER) status. It is increasingly recognized that the immune system, especially adaptive immune cells, has a large influence on the prognosis of breast cancer [5], [6]. The impact of adaptive cellular immune response, represented by CD8+ T cells, was studied most intensely. Many studies found that CD8+ T cells were associated with good prognosis [7]–[9]. Though the favourable impact of CD8+ T cells has been substantiated by these studies, the role of the humoral system, represented by B cells/plasma cells was acknowledged only recently [10]–[13].

In this regard, a recent study reported that 55% out of the 1470 breast cancers were infiltrated by B cells [11]. Wang et al. showed that an immune response against tumour-derived antigens led to the maturation and differentiation of B cells and that immunoglobulin (Ig) G was the dominant isotype in invasive breast tumours [14]. Accordingly, several studies showed that B cells were significantly associated with better prognosis [10]–[12]. Despite these findings, some experimental studies pointed to an adverse role of B cells suggesting that B cells may under certain conditions also stimulate progression of breast cancer [15]–[18].

Utilizing microarray-based gene-expression analysis, we could show that a stronger expression of a B cell metagene was associated with improved survival in node-negative breast cancer [10]. Building on these results, we described that immunoglobulin Kappa C (IGKC), a single gene of this B cell metagene, was found to be a representative marker and showed a favourable metastasis-free survival (MFS) in breast cancer both at the ribonucleic acid (RNA) and at the protein level [12]. Based on these encouraging findings, we examined in the present study the impact of immunohistochemically detected IGKC for disease-free survival (DFS) and breast cancer-specific overall survival (OS) in node-negative breast cancer patients who did not receive systemic therapy in the adjuvant setting. We also analysed the prognostic impact of IGKC in subgroups according to estrogen receptor expression as well as in luminal A and luminal B carcinomas.

**Figure 1:**
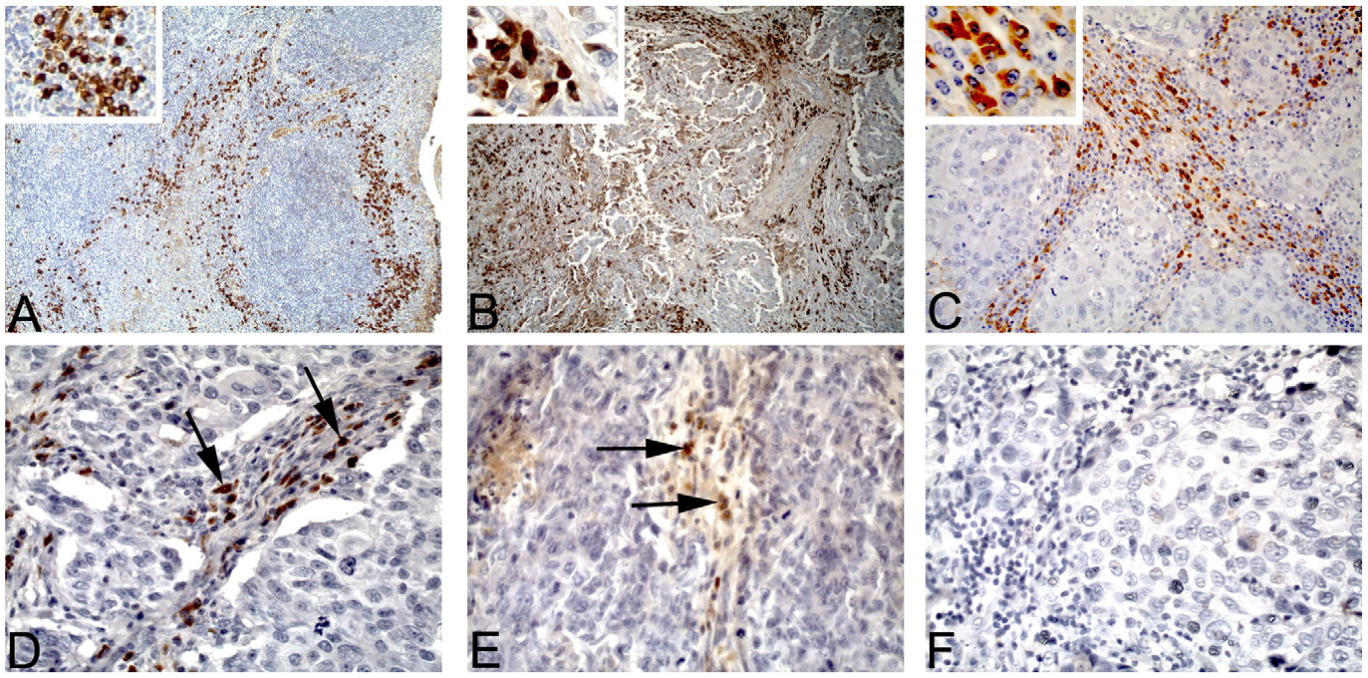
Representative examples of IGKC immunostaining in a positive control and breast cancer. (A) Normal human tonsil tissue, strong IGKC positive infiltrate was mainly distributed in the parafollicular area (original magnification: 100-fold; inset: 400-fold). (B) Strong IGKC positive infiltrate in invasive breast cancer (IGKC positive infiltrate score: 3+) (original magnification: 100-fold; inset: 400-fold). (C) Strong IGKC positive infiltrate in medullary breast cancer (IGKC positive infiltrate score: 3+) (original magnification: 200-fold; inset: 400-fold). (D) Moderate IGKC positive infiltrate (IGKC positive infiltrate score: 2+) (original magnification: 400-fold). (E) Weak IGKC positive infiltrate (IGKC positive infiltrate score: 1+) (original magnification: 400-fold). (F) IGKC negative breast cancer (IGKC positive infiltrate score: 0) (original magnification: 400-fold).

**Table 1:** Clinicopathological characteristics of all patients (n = 335).

Characteristics	Number	%
Age at diagnosis		
<50 years	84	25.1
≥50 years	251	74.9
pT stage		
pT_1_	222	66.3
pT_2_	110	32.8
pT_3_	3	0.9
Histological grade		
G I	87	26.0
G II	183	54.6
G III	65	19.4
Estrogen receptor status		
Negative	64	19.1
Positive	271	80.9
Progesterone receptor status		
Negative	92	27.5
Positive	243	72.5
HER-2 status		
Negative	290	86.6
Positive	45	13.4
Ki-67 expression		
Low	235	70.1
High	87	26.0
Missing	13	3.9
IGKC positive infiltrate score		
0	79	23.6
1+	96	28.7
2+	43	12.8
3+	117	34.9
IGKC expression		
Low	175	52.3
High	160	47.7
IGKC status		
Negative	79	23.6
Positive	256	76.4
Death	92	27.5
Due to cancer	45	13.4
Unrelated to cancer	41	12.3
Unknown causes	6	1.8
Surviving	243	72.5
Relapse		
Yes	78	23.3
No	257	76.7

HER-2 human epidermal growth factor receptor 2.

## Methods

### Study Patients

Our initial study cohort included 410 consecutive lymph node-negative breast cancer patients not treated in the adjuvant setting. The tumor size was pT_1_ to pT_3_ and there was adequate follow-up information of patients who were treated at the Department of Obstetrics and Gynaecology of Johannes Gutenberg University Mainz between the years 1985 and 2001. Of these 410 patients, paraffin blocks with tumour tissue for IGKC immunohistochemistry (IHC) were available of 335 individuals who were analysed in this study. All these patients were treated by surgical tumour resection and did not receive any systemic adjuvant therapy. pT stage was collected from the pathology report of the Gynaecological Pathology Division. From the breast cancer database [19], results of age at diagnosis, histological grade, estrogen receptor (ER) status, progesterone receptor (PR) as well as Ki-67 and human epidermal growth factor receptor 2 (HER-2) status were obtained. Briefly, serial sections of formalin-fixed and paraffin-embedded tumor tissues were stained with monoclonal ER antibodies (clone 1D5, Dako, Glostrup, Denmark), monoclonal progesterone receptor (PR) antibodies (clone PgR 636, Dako, Glostrup, Denmark), monoclonal Ki-67 antibodies (clone MIB-1, Dako, Glostrup, Denmark) as well as polyclonal HER-2 antibodies (A0485, Dako, Glostrup, Denmark). HER-2 was scored from 0 to 3+ according to the well-published manufacturer’s instructions. HER-2 3+ tumors were considered HER-2 positive. All HER-2 2+ cases were confirmed by Fluorescence in-situ hybridization (FISH) using a dual-color probe (DakoCytomation) containing a spectrum orange-labeled HER-2 gene (17q11.2-q12) probe and a spectrum green-labeled centromere control for chromosome 17 (17p11.1-q11.1). HER-2 tumors with 2+ HER-2 amplification were finally considered HER-2 positive. ER and PR expression was analysed as percentage of all tumor cells and any nuclear expression >0 was considered positive. Ki67 expression of more than 20% was considered as high expression and a percentage ≤20% was defined as low expression [20]. Luminal A and luminal B type carcinomas were defined according to Goldhirsch et al. [21]. Briefly, ER and/or PR positive carcinomas were defined as luminal A if they were both HER2 negative and well or moderately differentiated. Conversely, ER and/or PR positive carcinomas were classified as luminal B if they were either HER2 positive or poorly differentiated. Among 410 breast cancer patients, 224 (55%) patients were treated with breast conserving surgery followed by irradiation and 185 (45%) with modified radical mastectomy. We only included node-negative breast cancer patients with pT_1–3_ tumours without any evidence of metastatic disease at the time of surgery. The median age at diagnosis of the patients was 60 years (range 33 to 91 years). We documented death from cancer or from other reasons unrelated to breast cancer and recurrence of disease, which include metastasis, local relapse and secondary tumours. The mean follow-up time was 152 months. 45 (13.4%) patients died from breast cancer, 41 (12.3%) patients died from other diseases unrelated to breast cancer, 6 (1.8%) patients died from unknown causes, 243 (72.5%) patients were alive and 78 (23.3%) patients suffered from recurrent disease. The patients dying from other reasons were censored from their survival statistics analysis at their date of death. The study was approved by the ethical review board of the medical association of Rhineland-Palatinate. The manuscript was prepared in agreement with the reporting recommendations for tumor marker reporting studies [22].

**Figure 2:**
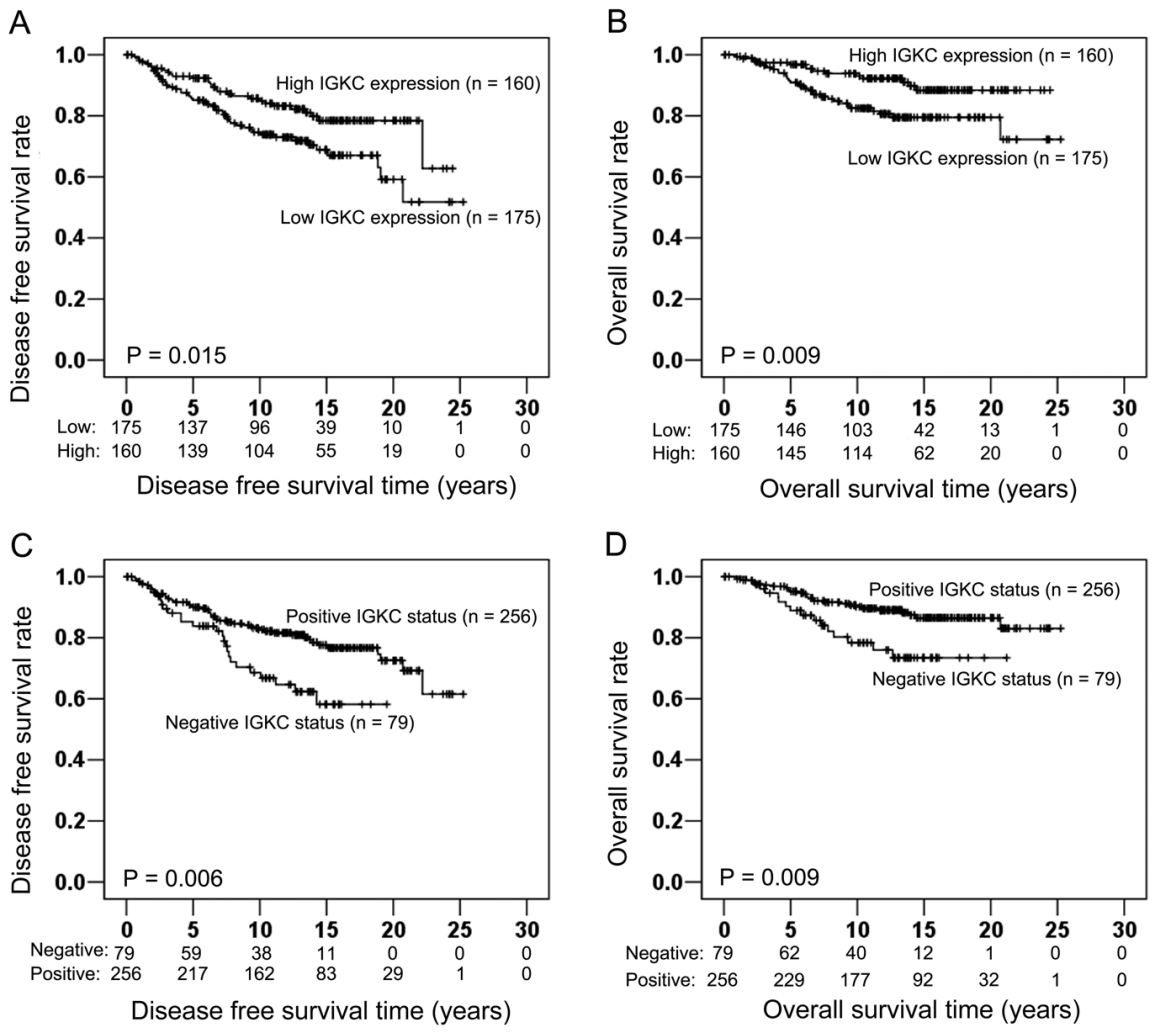
Association of IGKC expression with prognosis in the entire cohort (n = 335). Kaplan Meier survival analysis illustrated that high IGKC expression was significantly associated with longer DFS (Log-rank test: P = 0.015; Fig. 2A) and longer OS (Log-rank test: P = 0.009; Fig. 2B). A comparable prognostic influence was seen when IGKC status was used for DFS (Log-rank test: P = 0.006; Fig. 2C) and OS (Log-rank test: P = 0.009; Fig. 2D), respectively.

**Table 2:** Cox regression analysis of IGKC expression for disease-free survival (DFS) in the entire cohort (n = 335).

Clinicopathological Characteristics	HR	95% CI	*P*
A. Univariate Cox analysis			
IGKC expression (low vs. high)	0.570	0.360–0.903	0.017
Age (<50 years vs. ≥50 years)	1.290	0.764–2.176	0.341
pT stage (≤2 cm vs. >2 cm)	1.354	0.862–2.127	0.188
Histological grade (G I and II vs. G III)	3.404	2.155–5.377	<0.001
ER status (negative vs. positive)	0.802	0.472–1.360	0.412
PR status (negative vs. positive)	0.759	0.473–1.216	0.251
HER-2 status (negative vs. positive)	2.282	1.360–3.827	0.002
Ki-67 expression a (low vs. high)	2.193	1.385–3.472	0.001
B. Multivariate Cox analysis			
IGKC expression (low vs. high)	0.504	0.315–0.805	0.004
Age (<50 years vs. ≥50 years)	1.206	0.704–2.065	0.495
pT stage (≤2 cm vs. >2 cm)	1.430	0.901–2.269	0.129
Histological grade (G I and II vs. G III)	3.617	2.197–5.954	<0.001
ER status (negative vs. positive)	1.394	0.660–2.941	0.384
PR status (negative vs. positive)	0.963	0.501–1.849	0.909
HER-2 status (negative vs. positive)	2.015	1.176–3.454	0.011

ER Estrogen receptor; PR Progesterone receptor; HER-2 human epidermal growth factor receptor 2; HR hazard ratio; 95%-CI 95%-confidence interval.

a The total number of available cases for Ki-67 expression in univariate Cox regression analysis is 322.

### Ethics Statement

The study was approved by the ethical review board of the medical association of Rhineland-Palatinate. Informed consent has been obtained and all clinical investigation has been conducted according to the principles expressed in the Declaration of Helsinki.

**Table 3:** Cox regression analysis of IGKC expression for overall survival (OS) in the entire cohort (n = 335).

Clinicopathological Characteristics	HR	95% CI	*P*
A. Univariate Cox analysis			
IGKC expression (low vs. high)	0.438	0.233–0.824	0.011
Age (<50 years vs. ≥50 years)	1.140	0.584–2.223	0.702
pT stage (≤2 cm vs. >2 cm)	1.744	0.971–3.134	0.063
Histological grade (G I and II vs. G III)	4.630	2.577–8.321	<0.001
ER status (negative vs. positive)	0.753	0.381–1.488	0.415
PR status (negative vs. positive)	0.849	0.452–1.597	0.613
HER-2 status (negative vs. positive)	2.520	1.301–4.881	0.006
Ki-67 expression a (low vs. high)	2.701	1.502–4.858	0.001
B. Multivariate Cox analysis			
IGKC expression (low vs. high)	0.375	0.197–0.713	0.003
Age (<50 years vs. ≥50 years)	1.097	0.551–2.182	0.793
pT stage (≤2 cm vs. >2 cm)	1.848	1.012–3.374	0.046
Histological grade (G I and II vs. G III)	5.206	2.766–9.801	<0.001
ER status (negative vs. positive)	1.202	0.413–3.504	0.736
PR status (negative vs. positive)	1.349	0.505–3.606	0.551
HER-2 status (negative vs. positive)	2.333	1.166–4.668	0.017

ER Estrogen receptor; PR Progesterone receptor; HER-2 human epidermal growth factor receptor 2; HR hazard ratio; 95%-CI 95%-confidence interval.

a The total number of available cases for Ki-67 expression in univariate Cox regression analysis is 322.

### Immunohistochemistry

Immunostaining was done on 4 µm thick sections according to standard procedures as previously described [14]. Briefly, serial sections of formalin-fixed and paraffin-embedded tumour tissue were subsequently deparaffinized using graded alcohol and xylene. Antigen retrieval reactions were performed in a steamer in citrate buffer of pH10 for 30 minutes. 3% H_2_O_2_ solution was applied to block endogenous peroxidase at room temperature for 5 minutes. Monoclonal IGKC antibodies (Clone KP-53; Santa Cruz Biotechnology Company, Santa Cruz, California, USA) in a dilution of 1∶100 was used to incubate with the tissue sections for 30 minutes at room temperature in a humidified chamber, followed by polymeric biotin–free visualization system (Envision™, DAKO Diagnostic Company, Hamburg, Germany) reaction for 30 minutes at room temperature. Then the sections were incubated with 3, 3-diaminobenzidine (DAB) (Envision™, DAKO Diagnostic Company, Hamburg, Germany) in a dilution of 1∶50 with substrate buffer for 5 minutes at room temperature and counterstained with Mayer’s haematoxylin solution for 5 minutes. All slides were mounted and then were evaluated under a Leica light microscope (Leica Microsystem Vertrieb Company, Wetzler, Germany) by two of the authors trained in histological and immunohistochemical diagnostics, unaware of the clinical outcome. All series included appropriate positive (tonsil) and negative (hepatocytes) controls, and all controls gave adequate results.

**Table 4:** Bivariate Cox analysis of IGKC expression with Ki-67 expression for disease-free survival (DFS) (A) and overall survival (OS) (B) (n = 322).

Clinicopathological Characteristics	HR	95% CI	*P*
A. Disease free survival (DFS)			
IGKC expression (low vs. high)	0.555	0.346–0.889	0.014
Ki-67 expression (low vs. high)	2.131	1.345–3.376	0.001
B. Overall survival (OS)			
IGKC expression (low vs. high)	0.466	0.248–0.877	0.018
Ki-67 expression (low vs. high)	2.626	1.460–4.725	0.001

HR hazard ratio; 95%-CI 95%-confidence interval.

### Evaluation of Immunostaining

Evaluation was performed as previously described [12]. Since only the total number of B cells, irrespective of location, was found to be associated with prognosis [11], a semi-quantitative scoring method similar to that used by other studies [23], [24] was employed to evaluate the intensity of IGKC positive infiltrate: 0, no IGKC positive infiltrate; 1+, weak IGKC positive infiltrate; 2+, moderate IGKC positive infiltrate; 3+, strong IGKC positive infiltrate. To dichotomize the patients, cases with IGKC score 0 and 1+ were considered as having low IGKC expression and cases with 2+ and 3+ as high IGKC expression, respectively. Additionally, we examined as IGKC status the differentiation between 0 (unequivocally negative) and positive (any staining, not regarding the extent). In case of disagreement of the results of two independent examiners the slides were re-examined and discussed at the microscope until a consensus was reached.

**Figure 3:**
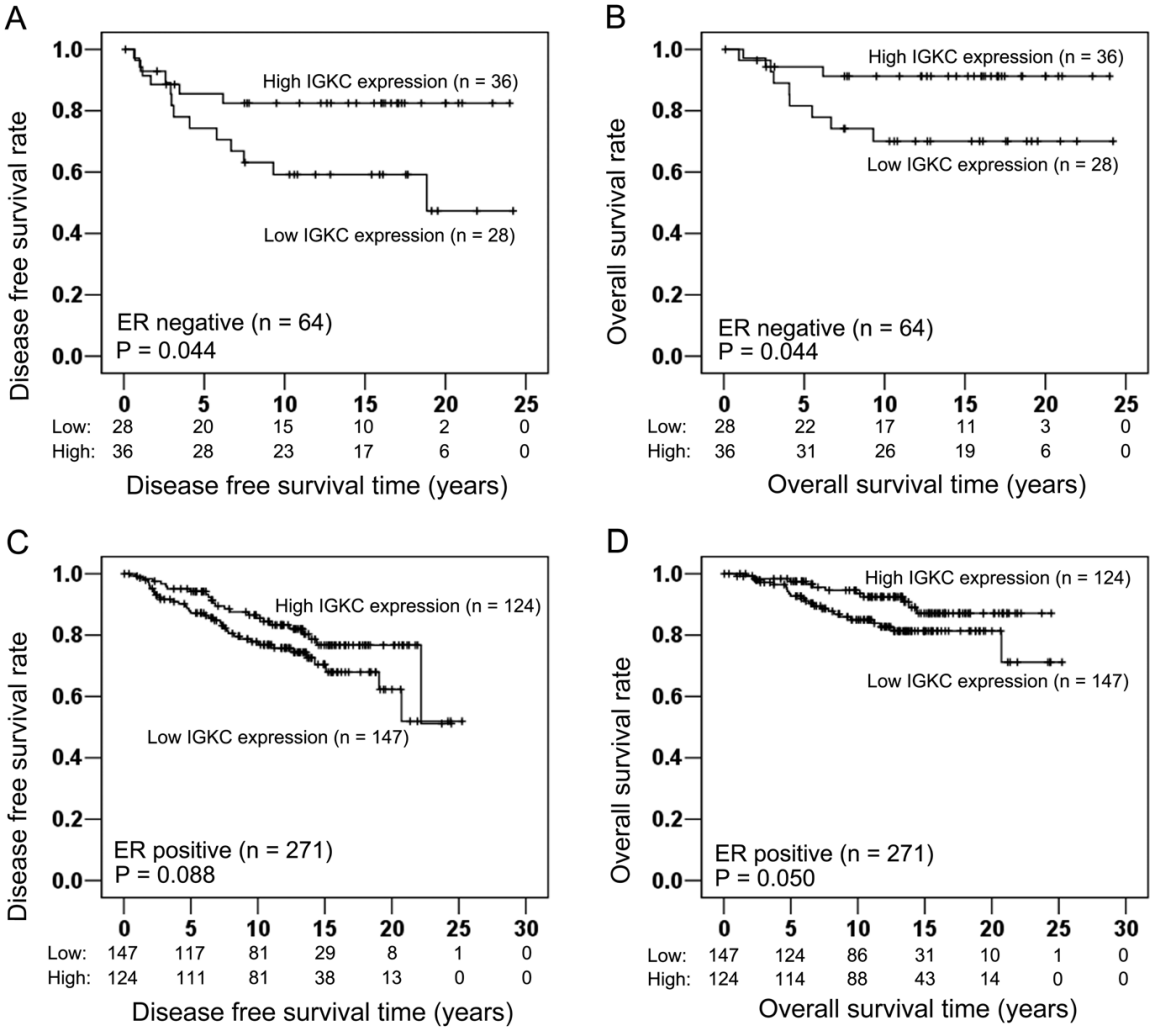
In ER negative carcinomas (n = 64), high IGKC expression had a significant association with longer DFS (Log-rank test: P = 0.044; Fig. 3A) and longer OS (Log-rank test: P = 0.044; Fig. 3B). (C, D) In ER positive carcinomas (n = 271), Kaplan Meier survival analysis showed that there was no significant association between IGKC and DFS (Log-rank test: P = 0.088; Fig.3C), and OS had a borderline association with IGKC (Log-rank test: P = 0.050; Fig.3D).

**Figure 4:**
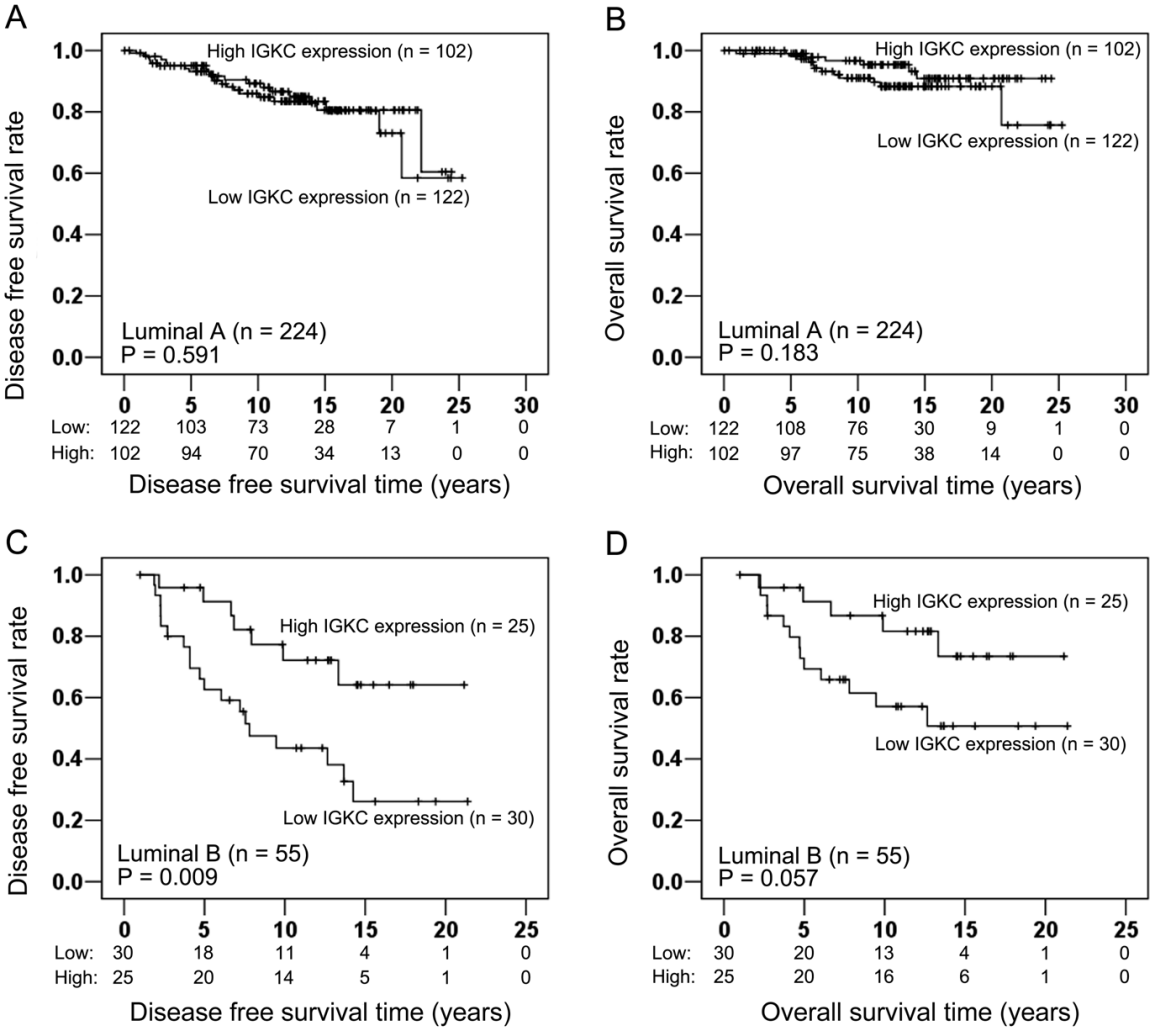
In luminal A carcinomas (n = 224), IGKC had no significant association with DFS (Log-rank test: P = 0.591; Fig. 4A) and OS (Log-rank test: P = 0.183; Fig. 4B). (C, D) In luminal B carcinomas (n = 55), Kaplan-Meier curves illustrated that high IGKC expression had significantly longer DFS than low IGKC expression (Log-rank test: P = 0.009; Fig. 4C). Moreover, OS also showed a borderline association with IGKC (Log-rank test: P = 0.057; Fig. 4D).

### Statistical Analysis

Survival rates were calculated according to the Kaplan-Meier method. Breast cancer-specific DFS was calculated from the diagnosis date to the date of recurrence including local relapse, distant metastasis, detection of the contra lateral breast cancer and death from cancer. Breast cancer-specific OS was computed from the date of diagnosis to the date of death from breast cancer. Patients who died of an unrelated cause were censored at the date of death. Survival was compared with the Log-rank test. Univariate and multivariate Cox analysis with proportional hazard regression model were employed to assess the impact of IGKC and other prognostic factors. Multivariate Cox survival analyses were done with inclusion. Dichotomization was done as follows: IGKC expression in low and high, age at diagnosis in <50 years and ≥50 years, pT stage in pT_1_ (≤2 cm) versus pT_2_ and pT_3_ (>2 cm), histological grade in G I and G II versus G III, ER status in negative and positive, PR status in negative and positive, HER-2 status in negative and positive, and Ki-67 expression in low and high. IGKC expression in the whole cohort as well as in ER positive, ER negative, luminal A and luminal B were assessed and Kaplan-Meier calculation, univariate and multivariate Cox analysis of IGKC expression for DFS and OS were done. Correlations between IGKC expression, age at diagnosis, pT stage, histological grade, ER status, PR as well as HER-2 status and Ki-67 expression were analyzed using the Chi-Square test (likelihood quotient). All P values were two sided. Since no correction for multiple testing was done, all results were interpreted as explorative. All statistical analyses were done using the Statistical Package for the Social Science (SPSS) (SPSS Inc, version 15.0, Chicago, IL, USA).

**Table 5:** Correlation of IGKC expression with clinicopathological characteristics (n = 335).

Characteristics	IGKC expression		*P* *
	Low (%)	High (%)	
No. of patients	175 (52.2)	160 (47.8)	
Age at diagnosis			
<50 years	43 (12.8)	41 (12.3)	0.824
≥50 years	132 (39.4)	119 (35.5)	
pT stage			
≤2 cm	124 (37.0)	98 (29.3)	0.063
>2 cm	51 (15.2)	62 (18.5)	
Histological grade			
G I and G II	143 (42.7)	127 (37.9)	0.589
G III	32 (9.5)	33 (9.9)	
Estrogen receptor status			
Negative	28 (8.4)	36 (10.7)	0.131
Positive	147 (43.9)	124 (37.0)	
Progesterone receptor status			
Negative	42 (12.6)	50 (14.9)	0.138
Positive	133 (39.7)	110 (32.8)	
HER-2 status			
Negative	152 (45.3)	138 (41.2)	0.871
Positive	23 (6.9)	22 (6.6)	
Ki-67 expression a			
Low	120 (37.3)	115 (35.7)	0.306
High	50 (15.5)	37 (11.5)	

* Correlation between variables was determined by Chi-Quare test.

HER-2 human epidermal growth factor receptor 2.

a The total number of available cases for Ki-67 expression is 322.

## Results

### Results of Immunohistochemistry

Established clinicopathological variables were assessed, including age at diagnosis, pT stage, histological grade, ER, PR as well as HER-2 status and Ki-67 expression (Table 1). IGKC expression was determined by immunohistochemistry (IHC) (Fig. 1). IGKC was found mainly in the tumour stroma. Using the IGKC scoring method [14], 79 (23.6%) patients were scored 0, 96 (28.7%) patients were scored 1+, 43 (12.8%) patients were scored 2+ and 117 (34.9%) patients were scored 3+. In order to obtain a comparable size of the groups, we combined score 0 and 1+ as well as 2+ and 3+, respectively. Accordingly, 175 (52.3%) patients were considered to have low IGKC expression and 160 (47.7%) patients to have high IGKC expression, respectively. For comparison, we also showed Kaplan Meier plots for IGKC status negative (n = 79; 23.6%) vs. positive (n = 256; 76.4%) in the entire cohort of patients. Only low and high IGKC expression was used for further analysis of the prognostic relevance of IGKC.

### IGKC has Protective Impact on Prognosis of Node-negative Breast Cancer Patients

In the total patient series, IGKC expression (P = 0.017, HR = 0.570, 95% CI = 0.360–0.903; Table 2A) showed a statistically significant association with DFS in univariate Cox analysis. In addition, histological grade (P<0.001, HR = 3.404, 95% CI = 2.155–5.377; Table 2A), HER-2 status (P = 0.002, HR = 2.282, 95% CI = 1.360–3.827; Table 2A) and Ki-67 expression (P = 0.001, HR = 2.193, 95% CI = 1.385–3.472; Table 2A) also had statistically significant associations with DFS. Kaplan-Meier plots illustrate a protective impact of IGKC expression on DFS (Log-rank test: P = 0.015; Fig. 2A). A similar effect was seen when IGKC status was used (Log-rank test: P = 0.006; Fig. 2C) In the multivariate Cox regression model including age at diagnosis, pT stage, histological grade, ER as well as PR and HER-2 status, high IGKC expression was independently associated with improved DFS (P = 0.004, HR = 0.504, 95% CI = 0.315–0.805; Table 2B). Besides IGKC expression, histological grade (P<0.001, HR = 3.617, 95% CI = 2.197–5.954; Table 2B) and HER-2 status (P = 0.011, HR = 2.015, 95% CI = 1.176–3.454; Table 2B) were also independently associated with DFS.

Similarly as for DFS, also OS showed associations with IGKC expression (P = 0.011, HR = 0.438, 95% CI = 0.233–0.824; Table 3A), histological grade (P<0.001, HR = 4.630, 95% CI = 2.577–8.321; Table 3A), HER-2 status (P = 0.006, HR = 2.520, 95% CI = 1.301–4.881; Table 3A) and Ki-67 expression (P = 0.001, HR = 2.701, 95% CI = 1.502–4.858; Table 3A) in the univariate Cox analysis. Furthermore, Kaplan Meier survival analysis visualized a strong difference in OS time between patients with low and high IGKC expression (Log-rank test: P = 0.009; Fig. 2B). A prognostic significance of similar magnitude was seen when IGKC status was used (Log-rank test: P = 0.009; Fig. 2D). Performing multivariate Cox analysis adjusted for age at diagnosis, pT stage, histological grade, ER as well as PR and HER-2 status, high IGKC expression was associated with better OS independent of other prognostic factors (P = 0.003, HR = 0.375, 95% CI = 0.197–0.713; Table 3B). In this multivariate Cox regression model, also pT stage (P = 0.046, HR = 1.848, 95% CI = 1.012–3.374; Table 3B), histological grade (P<0.001, HR = 5.206, 95% CI = 2.766–9.801; Table 3B) and HER-2 status (P = 0.017, HR = 2.333, 95% CI = 1.166–4.668; Table 3B) were associated with OS.

Conducting bivariate Cox analysis, IGKC expression had statistically significant associations with DFS (P = 0.014, HR = 0.555, 95% CI = 0.346–0.889; Table 4A) as well as OS (P = 0.018, HR = 0.466, 95%CI = 0.248–0.877; Table 4B) independent of Ki-67 expression.

### Prognostic Significance of IGKC in Subgroups According to ER and Luminal Status

In ER negative carcinomas (n = 64), both DFS and OS were significantly associated with IGKC expression. Kaplan Meier plots showed that high IGKC expression was associated with longer DFS (Log-rank test: P = 0.044; Fig. 3A) and longer OS (Log-rank test: P = 0.044; Fig. 3B). IGKC was not associated with DFS in Kaplan Meier analysis in ER positive carcinomas (n = 271) (Log-rank test: P = 0.088; Fig. 3C). OS showed a borderline significant association with IGKC expression in Kaplan Meier analysis in ER positive carcinomas (Log-rank test: P = 0.050; Fig. 3D).

When we separated the hormone receptor positive patients in luminal A (n = 224) and luminal B (n = 55) we failed to detect any significant impact of IGKC on DFS (Log-rank test: P = 0.591; Fig. 4A) and OS (Log-rank test: P = 0.183; Fig. 4B) in luminal A type cancer. In contrast, IGKC was significantly associated with DFS (Log-rank test: P = 0.009; Fig. 4C) and showed a borderline association with OS (Log-rank test: P = 0.057; Fig. 4D) in luminal B carcinomas.

No significant correlations were found between IGKC expression and age at diagnosis (P = 0.824), pT stage (P = 0.063), histological grade (P = 0.589), ER status (P = 0.131), PR status (P = 0.138), HER-2 status (P = 0.871), and Ki-67 expression (P = 0. 306) (Table 5).

## Discussion

The significance of the immune system is increasingly noticed in breast cancer. Since different immune cell types may have different functions, it is necessary to analyse the impact of individual cell types on survival. Being aware of this problem, several studies focusing on cellular immune response were done and the protective impact of CD8+ T cell was confirmed [7]–[9]. The roles of B cells, however, remained elusive [10]–[13], [15]–[18].

By using principal component analysis to visualize the expression of several metagenes in breast cancer, our group identified and characterized several metagenes associated with biological motifs like B-cell, T-cell, proliferation and ER metagenes, respectively. We showed that the B-cell metagene was of pivotal importance as a prognostic factor in node-negative breast cancer [10]. A confirmatory study performed by Bianchini et al. [25] obtained comparable results. Since the B-cell metagene includes 60 genes, it is labour-intensive and costly to analyse thus precluding its use as a prognostic factor in the daily routine. To solve this problem, we investigated IGKC as a representative marker of the B-cell metagene. Its RNA expression was already found to be associated with a good prediction of metastasis free interval across several independent datasets [12]. Since prognostic factors that are routinely used in breast cancer like ER, PR, HER-2 or Ki-67 are measured using immunohistochemistry, analysing IGKC at the protein level would be more convenient. By immunostaining IGKC, our current study clearly highlighted its favourable prognostic role in breast cancer. Kaplan-Meier analysis, univariate and multivariate Cox analysis illustrated that a stronger expression of IGKC was significantly associated with improved breast cancer specific survival and DFS independent of other prognostic factors in the entire cohort of node-negative breast cancer patients. The prognostic impact of IGKC was especially strong in ER negative and luminal B breast cancers. Luminal A and luminal B are well defined intrinsic subtypes separating hormone receptor positive patients into two subgroups with distinct prognosis [26]. Even though gene array analysis was initially used to define these subtypes, a simplified classification using hormone receptor status, HER-2 status and histological grade of differentiation as proliferation marker has been adopted as a useful shorthand [21]. There was no association between IGKC and prognosis in luminal A carcinomas. However, IGKC had a strong prognostic impact in luminal B carcinomas. This is consistent with studies reporting that in ER positive carcinomas, the influence of the B-cell metagene was particularly strong in highly proliferating breast cancer [10], [25].

It is well described that over-expression of immune response genes was more often identified in ER negative as compared with ER positive breast cancer [27]. The study performed by Oh et al. [28] explained this phenomenon further. These authors found that highly proliferating breast cancer showed an association with an enhanced immune response leading to better prognosis in both ER positive and ER negative cancers. The proportions of highly proliferative cancer cells in these two subtypes, however, were different. According to their data, about 60% of ER negative cancers were highly proliferating while in ER positive cancers the proportion was only 17%. Accordingly, approximately 35% of ER positive cancers were slowly growing as compared to only 8% ER negative cancers. Interestingly, about 36% of ER negative cancers had highly active immune response. The proportion of ER positive cancers with high immune response was only 20%, therefore supporting the notion that ER might have an inhibitory effect on immune response. Low proliferative activity of ER positive breast carcinomas might lead to an attenuated immune response and hence to a comparatively poor prognosis. In the ER negative cancers, however, a higher proportion of highly proliferative cancer cells might result in a strong immune response as reflected by a strong IGKC positive infiltrate, and thus these ER negative cancers had a better survival. A similar association between proliferation and immune response applies to highly proliferating luminal B type carcinomas which show a strong influence of IGKC expression.

A potential weakness of our study is the rather small sample size of only 335 patients which might affect subgroup analysis due to variable statistical power between subgroups of differing size with varying numbers of events. A second shortcoming is the lack of an independent validation cohort of node-negative patients not treated in an adjuvant setting. A potential strength, though, is that this population allows for assessing the pure prognostic effect of a biomarker without potential predictive interaction.

In conclusion, our results demonstrate that IGKC is an independent prognostic factor in untreated node-negative breast cancer patients. The prognostic significance is most distinct in ER negative as well as in luminal B breast cancer. IGKC is thus a novel prognostic factor which lends itself to systematic testing in formalin-fixed, paraffin-embedded tissue. Furthermore, it underscores the importance of a naturally occurring humoral immune response against breast cancer.
